# Evidence that the Migration of the Northern Subpopulation of Pacific Sardine (*Sardinops sagax*) off the West Coast of the United States Is Age-Based

**DOI:** 10.1371/journal.pone.0166780

**Published:** 2016-11-16

**Authors:** Jenny McDaniel, Kevin Piner, Hui-Hua Lee, Kevin Hill

**Affiliations:** Fisheries Resources Division, Southwest Fisheries Science Center, National Marine Fisheries Service, National Oceanic and Atmospheric Administration, La Jolla, California, United States of America; Maurice Lamontagne Institute, CANADA

## Abstract

Analysis of fish movements has been an important area of study for fisheries ecology and population dynamics for decades. Pacific sardine, *Sardinops sagax*, along the west coast of the United States exhibit a well-defined large-scale seasonal migration. Larger and older fish are found in the northern reaches of their range during summer and contract to southerly offshore areas for spawning during spring. Because of the close correlation between fish size and age it has not yet been determined if movements are size- or age-based. Measuring spatial changes in the age structure conditioned on individual lengths was used to determine the roles of age versus length in the seasonal migration. *S*. *sagax* have a pattern of increasing age-at-length with seasonal northward movements and offshore movements for spawning. The pattern of increasing age-at-length with distance from the origin eliminates a solely length-based process of movement and supports age-based movement. Patterns in the size and age when fish first show migratory behaviors, coupled with the patterns observed during the spawning season, support a hypothesis that migratory behaviors are linked to age-based ontogenetic changes associated with maturation.

## Introduction

Fish movements and large-scale migrations are one of the most studied aspects of fisheries biology and ecology. Research has focused on descriptions of individual population migratory patterns [1, 2], environmental cues [2–4], methods of identifying pathways [5], and the evolutionary advantages of migratory behaviors [6]. Fish movement has been characterized as either small-scale and within habitat movement or large-scale and between habitat migration. Within habitat movement is thought to be a response to changing habitat conditions while large-scale movements are related to environmental conditions as well as adaptive behaviors such as feeding and ontogenetic changes, particularly reproduction [7].

While patterns and physical cues for movement have been well described for many migratory populations, the relative roles that the related biological processes of size and age play in migration is not as clear. The arguments for size-based migration involve the energetic costs and physical capacity of large and small fish to move, favoring larger fish for large-scale migrations [8, 9]. Age-based movements could be related to age-specific maturation and learned behaviors regarding schooling, optimal foraging, and spawning locations. For some species, such as Atlantic cod, *Gadus morhua*, there are multiple groups within a population which display different migratory patterns hypothesized to have been learned from older fish [10–12]. MacCall [13] concluded that the loss of “migratory knowledge” within a population could cause a change in fitness leading to stock decline.

Three discrete subpopulations of *S*. *sagax*, occur off the west coast of North America from Alaska to Baja California, Mexico [14–17]. The northern subpopulation is typically found from northern Baja California to British Columbia, Canada and spawns in a discrete unit off southern and central California in offshore waters [17]. The migratory pattern of the northern subpopulation of *S*. *sagax* in the California Current Ecosystem is well defined [18, 19]. Initial studies of *S*. *sagax* movement patterns off California date back to an expansive tagging program started in the mid-1930s which revealed that sardine movement occurs seasonally northward and subsequently returning southward along the west coast of North America [20–23]. Clark and Marr [24] noted that northward movement occurred in summer whereas southward movement most often occurred in the winter. Recent work has focused on the oceanic cues for the onset of seasonal migrations [19, 25–34]. Similarly, oceanic conditions have been used to identify subpopulations in regions of mixing [31–33, 35].

The current migratory theory is that *S*. *sagax* move in response to changes in oceanic conditions related to shifts in the location of suitable feeding and spawning habitat. In spring, sardine spawn in the offshore waters of southern and central California and subsequently disperse northward from California to Canada compressed longitudinally along the coast [19]. While the exogenous factors influencing movement have been studied, the biological processes responsible are not well understood. *S*. *sagax* migration has been linked to both size and age [36]. Because of the close correlation between fish size and age [37], it is unresolved whether initiation of seasonal movement is determined by length-based [9] or age-based [38] biological processes.

The implications of age- versus length-based movement go beyond ecological questions, but are important considerations for the applied assessment of exploited populations. Fisheries demographic analyses (i.e. stock assessments) often convert observed length composition data to catch-at-age using a length-at-age relationship [39]. However, age-based movements invalidate the use of a simple age-length transition matrix as the age distribution associated with a length-class depends on geographic area, season and the rate of age-based movement. Unbiased assessment model estimates of biomass will depend on incorporating the process of movement with the correct structure (age or length).

Despite decades of study and the importance of the question for assessing population health, our understanding of the biological processes responsible for movement remains incomplete. This paper attempts to build on past work by using a method of conditional analysis to understand if the biological processes behind the movement and resulting spatial patterns of the northern subpopulation of *S*. *sagax* are the result of size- or age- based movement.

## Materials and Methods

### Study area and seasonal strata

**Fig 1:**
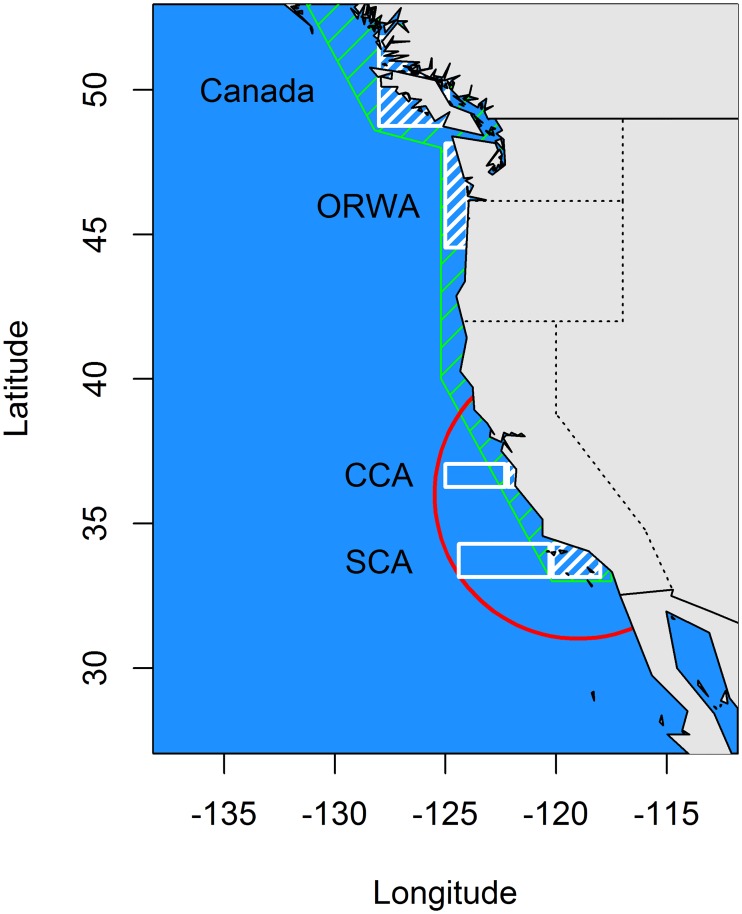
Range of the northern subpopulation of *S*. *sagax* with hypothesized migrations and study areas noted. Study areas: SCA onshore (southernmost white box with diagonal lines), SCA offshore (southernmost white box), CCA onshore (central white box with diagonal lines); CCA offshore (central white box), ORWA onshore (white box with diagonal lines off of the northwestern United States), CANADA (white box with diagonal lines off of Vancouver Island, British Columbia, Canada). Summer northward (green polygon with diagonal lines) and spring offshore migrations (red line) are noted.

This study was designed around the current theory that a segment of the northern subpopulation of *S*. *sagax* migrates northward along the coast after spawning and subsequently contracts southward to central and southern California. In spring, a segment of the subpopulation migrates to offshore waters to spawn (Fig 1). Quarters 3 (July-September) and 4 (October-December) represent the seasons of northward range expansion. Quarter 2 (April-June) represents the latitudinal contracted period with offshore spawning migrations.

Latitudinal range (Fig 1) was divided into four discrete areas: 1) southern California (SCA, latitude: 33°15’ to 34°30’N), 2) central California (CCA, latitude: 36°27’N to 37°06’N), 3) the Columbia River basin at the border of Oregon and Washington (ORWA, latitude: 44°54’N to 48°15’N), and 4) Canada (CANADA, latitude: 48°15’N to 51°48’N). Discrete boundaries are used because fisheries data are collected by port and not by fishing locations. Longitudinal (onshore-offshore) areas are defined as: 1) offshore SCA (longitude: west of 120°15’W), 2) offshore CCA (west of 122°18’W), and 3) onshore areas are found eastward. Both the SCA and CCA were analyzed for offshore movement because the center of spawning potentially occurs in either or both areas depending on the annual location of appropriate oceanic conditions.

### Sample collection

The study relies on random samples of paired age and length measurements, which were taken from both commercial fishery and trawl surveys. Commercial fisheries in all areas operated onshore and therefore offshore samples during spawning were taken from the trawl survey off of the United States which operates to the maximum limit of the offshore population distribution.

Sample data were obtained directly from state and federal agencies including CDFW, ODFW, WDFW, DFO, and NOAA. Commercial fishery samples were collected via state and federal fishery monitoring programs that sample licensed commercial fishing vessels at the ports. Trawl survey samples were collected under scientific research take permits obtained from the above mentioned agencies.

#### SCA and CCA commercial samples

Pacific sardine were collected by the California Department of Fish and Wildlife (CDFW) from southern and central California commercial fishing ports. SCA ports included San Pedro, CA, Terminal Island, CA, and Ventura, CA, while CCA ports included Monterey, CA and Moss Landing, CA. Biological information for each fish, including standard length, weight, sex, and maturity, was recorded. Sagittal otoliths were removed, cleaned, and stored dry.

Fish ages were determined following a standardized ageing method that was documented by Yaremko [40]. Otolith pairs were submerged in a dish of distilled water and placed on a black background sulcus side down. Otoliths were viewed with a stereomicroscope at 12-25x magnification with reflected light. An annulus was defined as “the interface between an inner translucent growth increment and the successive outer translucent growth increment”. Since the majority of spawning is believed to occur during the summer, a July 1^st^ birthdate was assumed for all fish hatched during a calendar year. Therefore, for fish caught prior to the July 1^st^ birthday, the most distal pair of opaque and translucent bands were not counted [40].

#### ORWA

Pacific sardine paired age-length samples collected by the Oregon Department of Fish and Wildlife (ODFW) and the Washington Department of Fish and Wildlife (WDFW) were used to characterize the Columbia River region. Samples were combined from both states because the fisheries operate in the same area around the mouth of the Columbia River [41]. Catches are landed and sampled in the ports of Astoria, OR and Westport, WA. Biological information and sagittal otoliths were collected similar to those described for SCA and CCA.

Fish ages were determined following the [40] standardized ageing method described above with the following slight modifications: (1) otolith pairs were submerged in either distilled water or ethanol, depending on reader preference; and (2) no birthdate assumption was made. As such, the most distal pair of opaque and translucent bands were counted as annuli. Since annuli counts were not adjusted for birthday, the annuli count was equal to the age of the fish. For fish collected after July 1^st^ assumed average birthday(start of quarter 3), this difference with Yaremko [40] is expected to have no impact on age assignment.

#### Canada

Pacific sardine paired age-length samples collected from Canada’s Department of Fisheries and Oceans (DFO) commercial and survey vessels were used to characterize the most northward reaches of the summer range expansion. Samples were collected from the United States and Canada border northward off of British Columbia, Canada. Biological information and sagittal otoliths were collected and aged using methods similar to those described for all other regions [42].

#### Fishery-independent trawl survey

To aid in the evaluation of onshore-offshore patterns of size and age, additional paired samples were taken from the Southwest Fisheries Science Center/National Oceanic and Atmospheric Administration (SWFSC/NOAA) trawl surveys. Biological information and sagittal otoliths were collected using the same methods as were used for the fishery samples. Otoliths were aged in accordance with the assumption of a July 1^st^ birthday for all fish [40].

### Conditional analysis

The general theory of conditional analysis is an extension from the analytical treatment of paired age-length data developed to estimate growth [38, 43, 44]. Because variability in the age-length relationship is a part of the growth form, each age contains a distribution of lengths associated with that age, and vice versa. Paired age-length samples can be analyzed by conditioning the observed distribution of one measurement (age or length) on a discrete unit of the other correlated measurement. Age- or length-based processes (sampling and biological) intervening between sample collection and the entire population’s age or size structure will affect the conditional distribution differently depending on: (1) the structure of the intervening process (age- or length-based); and (2) whether the data is conditioned on age or length. In this example, a solely length-based process of movement (higher proportion of fish moving with increasing length) would not be expected to affect the age distribution at an individual length (age conditioned on length) across the entire geographic range. However, if movements are age-based (higher proportion of fish moving with increasing age), the age distribution for individual lengths would be altered with distance from the origin. Similarly, a solely age-based process of movement would not affect the observed length distribution at-age (length conditioned on age) across geographic areas, but non-random length-based movement would affect the length distribution at-age in successive areas.

The study used the mean age at-length or mean length at-age to characterize changes in the distribution of ages or lengths by area. Bins with low sample sizes when conditioning on length (≤10 samples) or age (≤20 samples) were removed to avoid small sample bias. Commercial samples from 1981–2010 were used to examine north, south, and onshore areas. Trawl survey samples (2004 to 2010) were used to characterize quarter 2 offshore areas of the SCA and CCA. Detailed sample size information by age bin and length bin is provided in S1 and S2 Tables, respectively.

### Generalized linear model

A generalized linear model (GLM) assuming a normal error distribution for age or length was used. The estimated mean age or length is a linear function of fixed effects and interactions. A step-wise GLM procedure was used to determine the set of systematic effects and interactions that significantly explained the observed variability of ages or lengths using the Chi-square test. The marginal mean age-at-length by area and marginal mean length-at-age by area were calculated using least square means from the final model.

Mean age-at-length is represented by factors of year, length bin, area, and length and area interaction as:

$$Age_{ijk}=\mu +Year_{i}+Length_{j}+Area_{k}+Length_{j}\times Area_{k}+\varepsilon_{ijk}$$ (1)

where *μ* is overall mean, *Year*_*i*_ is effect of year *i*, *Length*_*j*_ is effect of length bin *j*, *Area*_*k*_ is effect of area *k*, *Length*_*j*_ × *Area*_*k*_ is interaction term between length bin *j* and area *k*, and *ε*_*ijk*_ is error term with *Normal*(0, *σ*^2^). For latitudinal movement *i* = 1981–2010, *j* = length bin 10–26 cm, *k* = SCA, CCA, ORWA, and CANADA. For onshore-offshore movement *i* = 2004–2010, *j* = length bin 10–26 cm, *k* = Onshore and Offshore.

Mean length-at-age is represented by factors of year, age bin, area, and age and area interaction as:

$$Length_{ijk}=\mu +Year_{i}+Age_{j}+Area_{k}+Age_{j}\times Area_{k}+\varepsilon_{ijk}$$ (2)

where *μ* is overall mean, *Year*_*i*_ is effect of year *i*, *Age*_*j*_ is effect of age bin *j*, *Area*_*k*_ is effect of area *k*, *Age*_*j*_ × *Area*_*k*_ is interaction term between age bin *j* and area *k*, and *ε*_*ijk*_ is error term with *Normal*(0, *σ*^2^). For latitudinal movement *i* = 1981–2010, *j* = age 0–12, *k* = SCA, CCA, ORWA, and CANADA. For onshore-offshore movement *i* = 2004–2010, *j* = age 0–7, *k* = Onshore and Offshore.

## Results

### Northward range expansion

**Fig 2:**
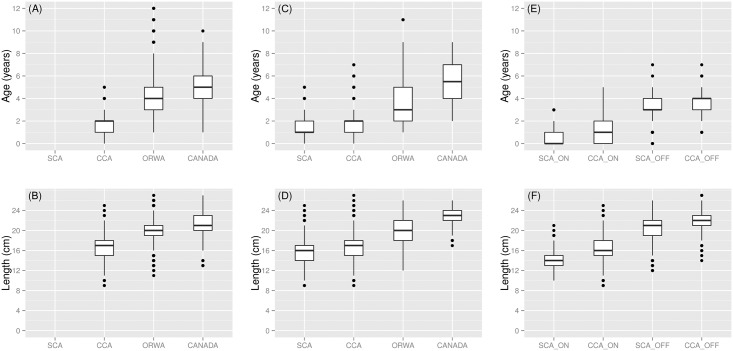
Boxplots of age and length by latitude during northern expansion for quarter 3 (July-September, 1981–2010) and quarter 4 (October-December, 1981–2010) and onshore-offshore during spawning (quarter 2, 2004–2010). (A) Quarter 3 age by latitude; and (B) length by latitude during the period of northern expansion. (C) Quarter 4 age by latitude; and (D) length by latitude during the period of northern expansion. (E) Quarter 2 age by onshore-offshore; and (F) length by onshore-offshore during spawning in central and southern California. The horizontal line in the box represents the mean, the box represents 25th and 75th percentiles, and the dots are outside 1.5 times the interquartile range above the upper quartile and below the lower quartile.

**Fig 3:**
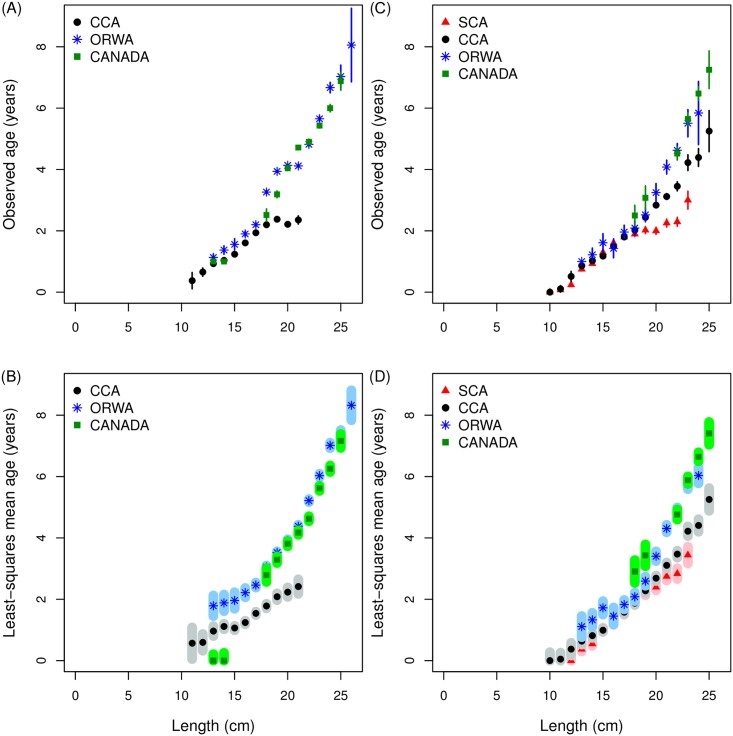
Observed mean age-at-length and least-squares mean age-at-length for quarter 3 (July-September) and quarter 4 (October-December) from 1981–2010. (A) Quarter 3 observed mean age-at-length; and (B) least-squares mean age-at-length for central California (CCA, ●), the Columbia River basin (ORWA, *), and Canada (CANADA, ■); (C) Quarter 4 observed mean age-at-length; and (D) least-squares mean age-at-length for Southern California (SCA, ▲), central California (CCA, ●), the Columbia River basin (ORWA, *), and Canada (CANADA, ■) where the error bars in the upper panels indicate the 95% confidence intervals of the observations and shaded areas in the lower panels represent 95% confidence intervals of the predictions.

*S*. *sagax* are both larger and older with increasing northward distance from SCA (Fig 2). Observed mean age-at-length increased with northward distance from SCA (Fig 3). Model prediction of mean age-at-length (Table 1) which accounted for year effects showed the same pattern as the aggregated data (Fig 3). The pattern of increasing mean age-at-length begins at 18–20 cm which corresponds to fish that have reached age 2. The difference in mean age-at-length between northern and southern areas is approximately 2 years at 20 cm and increases with increasing length.

**Table 1:** Analysis of deviance table for age-at-length and length-at-age from generalized linear models.

Quarter	Factor	SS	Df	F	Pr (>F)
3	Response: Age				
3	Year	7581	14	490.663	<0.0001
3	Length	21453	15	1295.883	<0.0001
3	Spatial	1782	2	807.233	<0.0001
3	Length:Spatial	430	17	22.945	<0.0001
3	Residuals	32960	29864		
3	Response: Length				
3	Year	23310	14	1141.651	<0.0001
3	Age	31158	12	1780.379	<0.0001
3	Spatial	5952	2	2040.536	<0.0001
3	Age:Spatial	547	11	34.075	<0.0001
3	Residuals	43630	29916		
4	Response: Age				
4	Year	1088.6	22	118.02	<0.0001
4	Length	5551.2	15	882.63	<0.0001
4	Spatial	430.2	3	341.97	<0.0001
4	Length:Spatial	249.5	29	20.52	<0.0001
4	Residuals	4862.9	11598		
4	Response: Length				
4	Year	14382.3	22	393.479	<0.0001
4	Age	22718.2	8	1709.226	<0.0001
4	Spatial	1157.4	3	232.213	<0.0001
4	Age:Spatial	745.6	12	37.399	<0.0001
4	Residuals	19277.7	11603		
2	Response: Age				
2	Year	135.33	6	71.3186	<0.0001
2	Length	1537.04	16	303.7497	<0.0001
2	Spatial	143.43	3	151.1762	<0.0001
2	Length:Spatial	30.83	22	4.4317	<0.0001
2	Residuals	1988.98	6289		
2	Response: Length				
2	Year	559	6	47.282	<0.0001
2	Age	9620.1	5	976.494	<0.0001
2	Spatial	917.2	3	155.174	<0.0001
2	Age:Spatial	685.7	8	43.504	<0.0001
2	Residuals	12474.1	6331		

Quarters 3 and 4 capture the period of northern expansion while quarter 2 captures the period of spawning in central and southern California.

**Fig 4:**
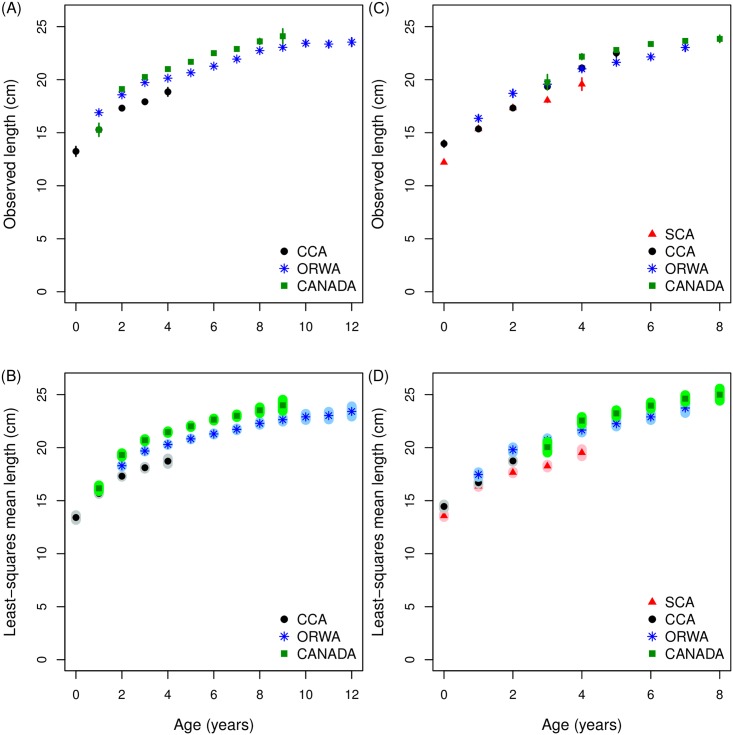
Observed mean length-at-age and least-squares mean length-at-age for quarter 3 (July-September) and quarter 4 (October-December) from 1981–2010. (A) Quarter 3 observed mean length-at-age; and (B) least-squares mean length-at-age for central California (CCA, ●), the Columbia River basin (ORWA, *), and Canada (CANADA, ■); (C) Quarter 4 observed mean length-at-age; and (D) least-squares mean length-at-age for Southern California (SCA, ▲), central California (CCA, ●), the Columbia River basin (ORWA, *), and Canada (CANADA, ■) where the error bars in the upper panels indicate the 95% confidence intervals of the observations and shaded areas in the lower panels represent 95% confidence intervals of the predictions.

In contrast with age conditioned on length, mean length-at-age by area was not strongly patterned with increasing northward distance (Fig 4). In particular, no obvious change to the pattern of mean length-at-age by area with increasing age (Table 1, Fig 4).

### Offshore movement during spawning

**Fig 5:**
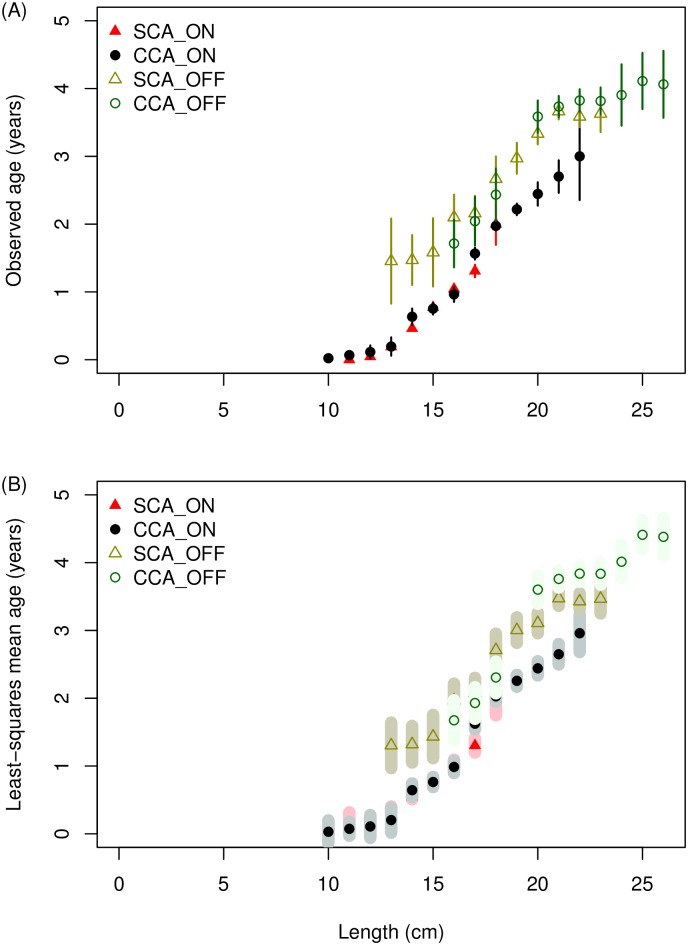
Observed mean age-at-length and least-squares mean age-at-length for quarter 2 (April-June), 2004–2010. (A) Observed mean age-at-length; and (B) least-squares mean age-at-length from the spawning areas of Southern California onshore (▲), Southern California offshore (Δ), central California onshore (●) and central California offshore (○), where the error bars in the upper panel indicate the 95% confidence intervals of the observations and shaded areas in the bottom panel represent 95% confidence intervals of the predictions.

*S*. *sagax* are larger and older offshore in the SCA and CCA during the spawning season (Fig 2). Model prediction of mean age-at-length (Table 1) which accounted for year effects showed the same pattern as the data (Fig 5). The pattern of increasing mean age-at-length offshore was observed for all lengths, which is a subtle difference from that shown in the northward expansion. An additional difference between spawning season (quarter 2) and quarters 3 and 4 was that during the spawning season there was no difference in mean age-at-length between the SCA and CCA (Fig 5).

**Fig 6:**
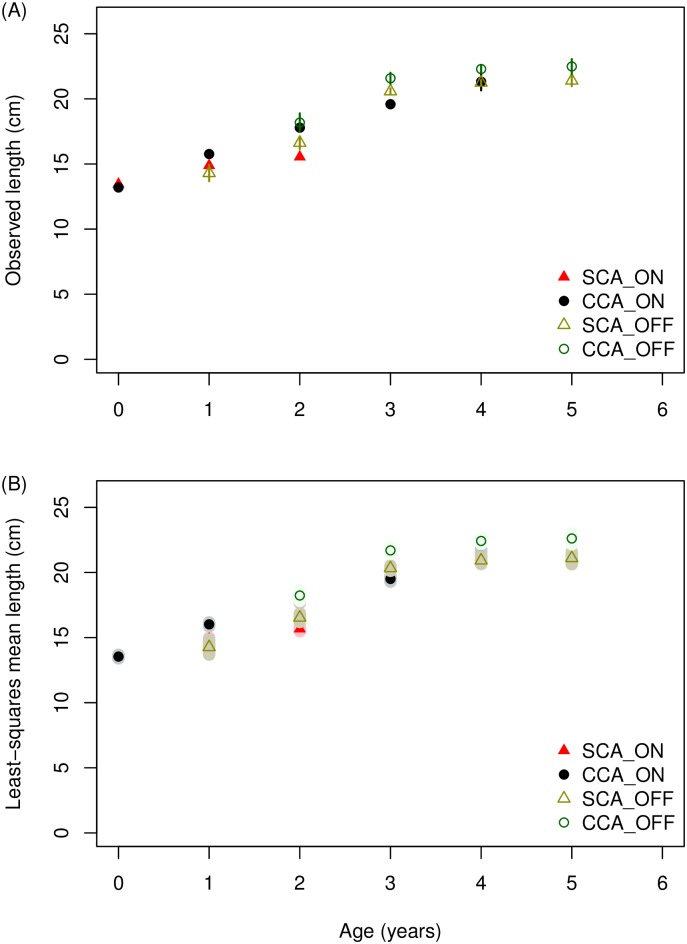
Observed mean length-at-age and least-squares mean length-at-age for quarter 2 (April-June, 2004–2010). (A) Observed mean length-at-age; and (B) least-squares mean length-at-age from the spawning areas of Southern California onshore (▲), Southern California offshore (Δ), central California onshore (●) and central California offshore (○), where the error bars in the upper panel indicate the 95% confidence intervals of the observations and shaded areas represent 95% confidence intervals of the predictions.

In contrast with age conditioned on length, mean length-at-age was not strongly patterned onshore-offshore (Fig 6). In particular, there was no obvious change to the pattern of mean length-at-age by onshore-offshore with increasing age. Model predictions and data were similar (Table 1, Fig 6).

## Discussion

The non-random patterns of mean age-at-length with increasing northward distance and offshore is consistent with a hypothesis of a strong intervening age-based process lying between the samples collected and the entire population age-structure. The most likely explanation is an age-based movement underlying the seasonal migrations of *S*. *sagax* [18, 19]. This interpretation is strengthened as this difference in mean age-at-length between SCA and CCA disappeared when the population contracted for spawning, indicative of mixing in the onshore and offshore areas. There is little evidence for length-based movements, not only because of the strong pattern when the data was conditioned on length, but the lack of pattern when the paired samples were conditioned on age. It is reasonable to expect that if movement was primarily controlled by a length-based process, a strong pattern of increasing mean length-at-age would have been apparent. In general, all results for length conditioned on age were far less clear, a result that would be expected with movement associated with age and not length.

The benefits of conditioning age on length as a method for controlling the effects of length-based sampling biases have been shown in studies estimating growth [38, 44, 45]. Fisheries analysis and population modelling has established that all removal methods have selection patterns that are a combination of both gear selectivity and availability. Gear selectivity (often called contact selectivity) is linked to capture probability typically based on fish size [46] while the availability component of a selectivity pattern is a measure of what specific segment of the total population’s age or size structure occurs in that spatial area. By accounting for length-based sampling biases, age conditioned on length controls for gear selectivity while giving insights into age availability. It should be noted that conditioning on length would not account for potentially confounding age-based behaviors that would make older fish more vulnerable to gears in a systematic way that aliased movement. However, these kinds of age-based behaviors would be atypical and require a pattern of behavior that seems unlikely.

In contrast to conditioning age on length, conclusions drawn from examining mean length conditioned on age is confounded by length-based processes. The effects of these length-based sampling biases on estimates of mean length have been well documented in the fisheries literature [44, 47, 48]. Spatial, temporal, and even individual vessel variability in gear selectivity cannot be discounted [38]. In addition, ecological differences in an individual unit of length (cm) are far less meaningful than a unit of difference in age (years) as each year contains many length units. Thus, conclusions drawn from area differences in mean length-at-age should be treated with caution and considered weaker evidence than that of age conditioned on the length. However, both lines of evidence present similar interpretations on the relative role of age versus length in the observed migratory patterns.

This study cannot rule out all potential causes of the area patterns in the paired data observed. Regional differences in exploitation levels with length-selective gears have been shown to alter regional size or age structure [49, 50]. However *S*. *sagax* are unlikely to incorporate a confounding cumulative regional effect due to the transient nature of the migratory fish. Another consideration of this study includes the assumption that ageing methods used to age all samples are equivalent. This assumption is likely reasonable given that ageing methods have been standardized and discrepancies minimized though ageing workshops held between the ageing laboratories [51]. Large differences in growth rates by region could also affect the results, but those differences would have to be very large and directly connected to which fish migrate to which areas each year. Finally, this work assumed a single migrating population. The existence of subpopulation structure could compromise our results.

A definitive explanation of the ecological motivation for age-based movements is beyond the scope of this paper, however a hypothesis that movements may be to be linked to the ontogenetic process of maturation is reasonable. The best evidence for the potential role of maturation as the biological cue for migration can be found in the offshore movements. In offshore spawning habitat every length class observed consisted of older fish on average than observed onshore. Considering that movement offshore is thought to be directly linked to spawning [17], this pattern provides strongest evidence of age-based movement’s relationship to maturation and spawning. *S*. *sagax* fully mature between the ages of 2–3 years [41] which corresponds to the ages associated with the first latitudinal differences in mean age-at-length identified in this study. The northward movement may not be directly linked to spawning but more likely relates to optimal feeding habitat in preparation for spawning the following spring. For simplicity of presentation, this paper combined male and female data, but the same patterns of conditional means were found for each sex separately and so the data were aggregated. This implies whatever the direct cause of age-based movement, it affects both sexes’ behaviors similarly at the gross spatial scales examined. However this discussion regarding the motivation for age-based migrations, as of now, is speculative and further research is warranted.

Conditional analysis of age-length pairs is another tool to understand the structure of the biological processes controlling population demography. The data requirements for conditional analysis are simple. However, the seasonal expansion-contraction movement patterns explored in this work made interpretation of the results much clearer than other potential movement patterns. With the discovery that movements are best explained as an age-based process, the use of a spatially explicit assessment models with age-based movements or alternatively modelling contact selectivity separate from age-based availability in single area models should be considered. The same considerations equally apply to research on basic life history parameters, such as growth and maturity, as estimates of these vital rates will be influenced by an age-based nature of movement. Studies aimed at estimating population level statistics need to be designed with consideration of the biological processes controlling spatial dynamics.

## Supporting Information

S1 Table Paired age-length measurements by area and quarter. Empirical mean age-at-length and standard deviation along with model estimated mean age and standard error are given. Length bins with 10 or fewer fish were not included in the analysis.
(PDF)

S2 Table The number of paired length-age measurements by area and quarter. Empirical mean length-at-age and standard deviation along with model estimated mean age and standard error are given. Length bins with 20 or fewer fish were not included in the analysis.
(PDF)
