# Supplementary material for: Evidence that the Migration of the Northern Subpopulation of Pacific Sardine (Sardinops sagax) off the West Coast of the United States Is Age-Based
Source: PLoS One. 2016 Nov 16;11(11):e0166780. doi: 10.1371/journal.pone.0166780 (PMC5112908; doi:10.1371/journal.pone.0166780)
Supplement: S1 Table — Empirical mean age-at-length and standard deviation along with model estimated mean age and standard error are given. Length bins with 10 or fewer fish were not included in the analysis. (PDF) [file pone.0166780.s001.pdf]

S1 Table. Paired age-length measurements by area and quarter.

		Model Estimation			Observed Values		
**Quarter**	**Area**	**Length (cm)**	**Mean Age (years)**	**SE**	**Mean Age (years)**	**sd**	**N**
2	SCA_ON	10					
2	SCA_ON	11	0.16	0.0788	0.00	0.0000	52
2	SCA_ON	12	0.19	0.0375	0.05	0.2268	244
2	SCA_ON	13	0.36	0.0210	0.19	0.4196	1000
2	SCA_ON	14	0.55	0.0193	0.46	0.5720	1098
2	SCA_ON	15	0.77	0.0217	0.77	0.5824	824
2	SCA_ON	16	1.04	0.0277	1.04	0.4486	513
2	SCA_ON	17	1.30	0.0528	1.31	0.5152	120
2	SCA_ON	18	1.98	0.1205	2.00	0.6901	22
2	SCA_ON	19					
2	SCA_ON	20					
2	SCA_ON	21					
2	SCA_ON	22					
2	SCA_ON	23					
2	SCA_ON	24					
2	SCA_ON	25					
2	SCA_ON	26					
2	CCA_ON	10	0.03	0.0852	0.02	0.1474	46
2	CCA_ON	11	0.07	0.0523	0.07	0.2512	134
2	CCA_ON	12	0.11	0.0864	0.11	0.3210	44
2	CCA_ON	13	0.20	0.0945	0.19	0.4014	36
2	CCA_ON	14	0.64	0.0510	0.63	0.7102	134
2	CCA_ON	15	0.76	0.0379	0.75	0.7103	259
2	CCA_ON	16	0.99	0.0458	0.96	0.7191	163
2	CCA_ON	17	1.62	0.0419	1.57	0.6431	205
2	CCA_ON	18	2.03	0.0399	1.97	0.5315	227
2	CCA_ON	19	2.26	0.0444	2.22	0.5761	175
2	CCA_ON	20	2.44	0.0534	2.45	0.9540	119
2	CCA_ON	21	2.65	0.0768	2.70	0.9056	57
2	CCA_ON	22	2.96	0.1416	3.00	1.2111	16
2	CCA_ON	23					
2	CCA_ON	24					
2	CCA_ON	25					
2	CCA_ON	26					
2	SCA_OFF	10					

		Model Estimation			Observed Values		
Quarter	Area	Length (cm)	Mean Age (years)	SE	Mean Age (years)	sd	N
2	SCA_OFF	11					
2	SCA_OFF	12					
2	SCA_OFF	13	1.30	0.1712	1.45	0.9342	11
2	SCA_OFF	14	1.32	0.1378	1.47	0.7174	17
2	SCA_OFF	15	1.43	0.1633	1.58	0.7930	12
2	SCA_OFF	16	1.97	0.1272	2.10	0.7182	20
2	SCA_OFF	17	2.08	0.1139	2.16	0.6245	25
2	SCA_OFF	18	2.71	0.1238	2.67	0.7303	21
2	SCA_OFF	19	3.00	0.0961	2.97	0.6636	35
2	SCA_OFF	20	3.11	0.0765	3.33	0.6713	72
2	SCA_OFF	21	3.47	0.0569	3.66	0.7038	134
2	SCA_OFF	22	3.43	0.0604	3.59	0.7826	99
2	SCA_OFF	23	3.47	0.1103	3.63	0.6877	27
2	SCA_OFF	24					
2	SCA_OFF	25					
2	SCA_OFF	26					
2	CCA_OFF	10					
2	CCA_OFF	11					
2	CCA_OFF	12					
2	CCA_OFF	13					
2	CCA_OFF	14					
2	CCA_OFF	15					
2	CCA_OFF	16	1.67	0.1510	1.71	0.6112	14
2	CCA_OFF	17	1.93	0.1187	2.04	0.8245	23
2	CCA_OFF	18	2.31	0.1190	2.43	0.8958	23
2	CCA_OFF	19					
2	CCA_OFF	20	3.60	0.1061	3.59	0.6278	29
2	CCA_OFF	21	3.76	0.0645	3.73	0.7171	83
2	CCA_OFF	22	3.84	0.0615	3.82	0.7971	91
2	CCA_OFF	23	3.84	0.0819	3.82	0.6974	49
2	CCA_OFF	24	4.01	0.1230	3.90	0.9952	21
2	CCA_OFF	25	4.41	0.1095	4.11	1.0500	27
2	CCA_OFF	26	4.38	0.1417	4.06	0.9287	16
3	CANADA	10					
3	CANADA	11					
3	CANADA	12					
3	CANADA	13	0.00	0.1076	1.00	0.0000	24

		Model Estimation			Observed Values		
**Quarter**	**Area**	**Length (cm)**	**Mean Age (years)**	**SE**	**Mean Age (years)**	**sd**	**N**
3	CANADA	14	0.00	0.1298	1.00	0.0000	27
3	CANADA	15					
3	CANADA	16					
3	CANADA	17					
3	CANADA	18	2.79	0.1146	2.52	0.9259	87
3	CANADA	19	3.29	0.0562	3.19	1.1244	388
3	CANADA	20	3.81	0.0439	4.04	1.0356	648
3	CANADA	21	4.17	0.0371	4.72	1.1122	938
3	CANADA	22	4.63	0.0417	4.90	1.1912	696
3	CANADA	23	5.62	0.0433	5.43	1.0388	649
3	CANADA	24	6.25	0.0542	6.00	1.2384	402
3	CANADA	25	7.16	0.1162	6.88	1.3471	83
3	CANADA	26					
3	SCA	10					
3	SCA	11					
3	SCA	12					
3	SCA	13					
3	SCA	14					
3	SCA	15					
3	SCA	16					
3	SCA	17					
3	SCA	18					
3	SCA	19					
3	SCA	20					
3	SCA	21					
3	SCA	22					
3	SCA	23					
3	SCA	24					
3	SCA	25					
3	SCA	26					
3	CCA	10					
3	CCA	11	0.57	0.2634	0.38	0.5000	16
3	CCA	12	0.59	0.1392	0.66	0.5148	58
3	CCA	13	0.96	0.0826	0.93	0.3177	171
3	CCA	14	1.11	0.0454	1.04	0.2843	610
3	CCA	15	1.06	0.0364	1.24	0.4780	925
3	CCA	16	1.24	0.0296	1.61	0.6035	1454

		Model Estimation			Observed Values		
**Quarter**	**Area**	**Length (cm)**	**Mean Age (years)**	**SE**	**Mean Age (years)**	**sd**	**N**
3	CCA	17	1.54	0.0286	1.94	0.6377	1693
3	CCA	18	1.78	0.0378	2.20	0.6322	1031
3	CCA	19	2.08	0.0569	2.38	0.6448	444
3	CCA	20	2.23	0.0785	2.21	0.5277	344
3	CCA	21	2.41	0.1209	2.35	0.6748	99
3	CCA	22					
3	CCA	23					
3	CCA	24					
3	CCA	25					
3	CCA	26					
3	ORWA	10					
3	ORWA	11					
3	ORWA	12					
3	ORWA	13	1.79	0.1716	1.13	0.3426	38
3	ORWA	14	1.88	0.1381	1.37	0.5219	59
3	ORWA	15	1.96	0.1380	1.56	0.7015	59
3	ORWA	16	2.21	0.0776	1.90	0.6388	197
3	ORWA	17	2.46	0.0424	2.20	0.8926	717
3	ORWA	18	3.05	0.0270	3.26	1.1475	1833
3	ORWA	19	3.52	0.0205	3.94	1.1328	4262
3	ORWA	20	3.91	0.0205	4.13	1.2185	4163
3	ORWA	21	4.38	0.0235	4.12	1.2532	3019
3	ORWA	22	5.22	0.0259	4.82	1.5232	2504
3	ORWA	23	6.03	0.0306	5.65	1.7601	1535
3	ORWA	24	7.02	0.0460	6.67	2.1011	582
3	ORWA	25	7.34	0.0896	7.03	2.2455	141
3	ORWA	26	8.32	0.2481	8.06	2.4125	18
4	CANADA	10					
4	CANADA	11					
4	CANADA	12					
4	CANADA	13					
4	CANADA	14					
4	CANADA	15					
4	CANADA	16					
4	CANADA	17					
4	CANADA	18	2.91	0.1893	2.50	0.5222	12
4	CANADA	19	3.43	0.1816	3.08	0.6405	13

		Model Estimation			Observed Values		
**Quarter**	**Area**	**Length (cm)**	**Mean Age (years)**	**SE**	**Mean Age (years)**	**sd**	**N**
4	CANADA	20					
4	CANADA	21					
4	CANADA	22	4.76	0.0837	4.52	0.868	65
4	CANADA	23	5.88	0.0612	5.65	1.1197	130
4	CANADA	24	6.64	0.0751	6.48	1.0089	82
4	CANADA	25	7.41	0.1888	7.25	0.9653	12
4	CANADA	26					
4	SCA	10	0.00	0.0857	0.00	0	46
4	SCA	11	0.00	0.0196	0.07	0.2632	188
4	SCA	12	0.00	0.0260	0.24	0.4274	226
4	SCA	13	0.37	0.0374	0.75	0.5718	421
4	SCA	14	0.55	0.0342	0.93	0.5022	511
4	SCA	15	1.01	0.0304	1.29	0.5715	659
4	SCA	16	1.33	0.0291	1.61	0.6949	674
4	SCA	17	1.55	0.0304	1.80	0.7416	620
4	SCA	18	1.93	0.0345	1.89	0.8301	418
4	SCA	19	2.21	0.0438	2.02	0.9062	259
4	SCA	20	2.39	0.0516	2.00	0.8059	195
4	SCA	21	2.75	0.0684	2.25	0.7063	118
4	SCA	22	2.83	0.0908	2.29	0.6	68
4	SCA	23	3.44	0.1391	3.00	0.7071	25
4	SCA	24					
4	SCA	25					
4	SCA	26					
4	CCA	10	0.00	0.1506	0.00	0	19
4	CCA	11	0.05	0.1088	0.11	0.3148	37
4	CCA	12	0.37	0.1057	0.51	0.5064	39
4	CCA	13	0.64	0.0575	0.86	0.3501	148
4	CCA	14	0.81	0.0363	1.02	0.2656	465
4	CCA	15	0.99	0.0284	1.18	0.4598	988
4	CCA	16	1.28	0.0272	1.49	0.6489	1112
4	CCA	17	1.58	0.0282	1.80	0.674	941
4	CCA	18	1.87	0.0308	2.02	0.6066	837
4	CCA	19	2.27	0.0348	2.44	0.6573	713
4	CCA	20	2.69	0.0443	2.84	0.7473	300
4	CCA	21	3.10	0.0481	3.12	0.7468	239
4	CCA	22	3.47	0.0574	3.45	0.9342	155

		Model Estimation			Observed Values		
**Quarter**	**Area**	**Length (cm)**	**Mean Age (years)**	**SE**	**Mean Age (years)**	**sd**	**N**
4	CCA	23	4.22	0.0800	4.22	1.0906	72
4	CCA	24	4.41	0.1041	4.39	0.9186	41
4	CCA	25	5.25	0.1889	5.25	1.0553	12
4	CCA	26					
4	ORWA	10					
4	ORWA	11					
4	ORWA	12					
4	ORWA	13	1.11	0.1754	1.00	0	14
4	ORWA	14	1.33	0.1180	1.22	0.6082	32
4	ORWA	15	1.72	0.1117	1.61	0.8711	36
4	ORWA	16	1.45	0.1437	1.43	0.6761	21
4	ORWA	17	1.83	0.0779	1.96	0.9722	76
4	ORWA	18	2.08	0.0717	2.09	1.0938	89
4	ORWA	19	2.59	0.0766	2.52	0.9949	77
4	ORWA	20	3.40	0.0808	3.25	1.2296	69
4	ORWA	21	4.31	0.0619	4.08	1.2896	128
4	ORWA	22	4.88	0.0554	4.63	1.4505	168
4	ORWA	23	5.75	0.0793	5.51	1.9012	73
4	ORWA	24	6.04	0.1315	5.84	2.4947	25
4	ORWA	25					
4	ORWA	26					

Empirical mean age-at-length and standard deviation along with model estimated mean age and standard error are given. Length bins with 10 or fewer fish were not included in the analysis.
