# Supplementary material for: Evidence that the Migration of the Northern Subpopulation of Pacific Sardine (Sardinops sagax) off the West Coast of the United States Is Age-Based
Source: PLoS One. 2016 Nov 16;11(11):e0166780. doi: 10.1371/journal.pone.0166780 (PMC5112908; doi:10.1371/journal.pone.0166780)
Supplement: S2 Table — Empirical mean length-at-age and standard deviation along with model estimated mean age and standard error are given. Length bins with 20 or fewer fish were not included in the analysis. (PDF) [file pone.0166780.s002.pdf]

**S2 Table. The number of paired length-age measurements by area and quarter.**

| | | Model Estimation | | | Observed Values | | |
|---|---|---|---|---|---|---|---|
| **Quarter** | **Area** | **Age (years)** | **Mean Length (cm)** | **SE** | **Mean Length (cm)** | **sd** | **N** |
| 2 | SCA_ON | 0 | 13.56 | 0.0412 | 13.45 | 1.0326 | 2042 |
| 2 | SCA_ON | 1 | 14.91 | 0.0424 | 14.88 | 1.1016 | 1603 |
| 2 | SCA_ON | 2 | 15.67 | 0.0946 | 15.54 | 1.4170 | 233 |
| 2 | SCA_ON | 3 | | | | | |
| 2 | SCA_ON | 4 | | | | | |
| 2 | SCA_ON | 5 | | | | | |
| 2 | SCA_ON | 6 | | | | | |
| 2 | SCA_ON | 7 | | | | | |
| 2 | CCA_ON | 0 | 13.54 | 0.0708 | 13.20 | 2.4005 | 482 |
| 2 | CCA_ON | 1 | 16.02 | 0.0761 | 15.77 | 1.7583 | 396 |
| 2 | CCA_ON | 2 | 17.78 | 0.0666 | 17.79 | 1.5449 | 546 |
| 2 | CCA_ON | 3 | 19.50 | 0.1094 | 19.59 | 1.3814 | 180 |
| 2 | CCA_ON | 4 | 21.30 | 0.2840 | 21.32 | 1.6258 | 25 |
| 2 | CCA_ON | 5 | | | | | |
| 2 | CCA_ON | 6 | | | | | |
| 2 | CCA_ON | 7 | | | | | |
| 2 | SCA_OFF | 0 | | | | | |
| 2 | SCA_OFF | 1 | 14.27 | 0.2956 | 14.30 | 1.4596 | 23 |
| 2 | SCA_OFF | 2 | 16.53 | 0.1698 | 16.63 | 2.0992 | 72 |
| 2 | SCA_OFF | 3 | 20.33 | 0.1028 | 20.58 | 1.6777 | 215 |
| 2 | SCA_OFF | 4 | 20.92 | 0.1437 | 21.24 | 1.2486 | 132 |
| 2 | SCA_OFF | 5 | 21.10 | 0.2426 | 21.41 | 1.3008 | 37 |
| 2 | SCA_OFF | 6 | | | | | |
| 2 | SCA_OFF | 7 | | | | | |
| 2 | CCA_OFF | 0 | | | | | |
| 2 | CCA_OFF | 1 | | | | | |
| 2 | CCA_OFF | 2 | 18.23 | 0.2126 | 18.18 | 2.3864 | 45 |
| 2 | CCA_OFF | 3 | 21.69 | 0.1344 | 21.58 | 2.3358 | 115 |
| 2 | CCA_OFF | 4 | 22.42 | 0.1134 | 22.29 | 1.7741 | 169 |
| 2 | CCA_OFF | 5 | 22.61 | 0.2272 | 22.49 | 1.7751 | 39 |
| 2 | CCA_OFF | 6 | | | | | |
| 2 | CCA_OFF | 7 | | | | | |
| 3 | CANADA | 0 | | | | | |
| 3 | CANADA | 1 | 16.17 | 0.1425 | 15.27 | 2.6858 | 74 |

| | | Model Estimation | | | Observed Values | | |
|---|---|---|---|---|---|---|---|
| **Quarter** | **Area** | **Age (years)** | **Mean Length (cm)** | **SE** | **Mean Length (cm)** | **sd** | **N** |
| 3 | CANADA | 2 | 19.31 | 0.0866 | 19.12 | 1.2613 | 214 |
| 3 | CANADA | 3 | 20.70 | 0.0566 | 20.26 | 1.3571 | 499 |
| 3 | CANADA | 4 | 21.46 | 0.0414 | 21.00 | 1.2467 | 952 |
| 3 | CANADA | 5 | 22.01 | 0.0376 | 21.69 | 1.2993 | 1175 |
| 3 | CANADA | 6 | 22.64 | 0.0515 | 22.50 | 1.3498 | 589 |
| 3 | CANADA | 7 | 23.01 | 0.0657 | 22.90 | 1.3439 | 362 |
| 3 | CANADA | 8 | 23.52 | 0.1404 | 23.61 | 1.2400 | 75 |
| 3 | CANADA | 9 | 23.99 | 0.2705 | 24.10 | 1.4832 | 20 |
| 3 | CANADA | 10 | | | | | |
| 3 | CANADA | 11 | | | | | |
| 3 | CANADA | 12 | | | | | |
| 3 | SCA | 0 | | | | | |
| 3 | SCA | 1 | | | | | |
| 3 | SCA | 2 | | | | | |
| 3 | SCA | 3 | | | | | |
| 3 | SCA | 4 | | | | | |
| 3 | SCA | 5 | | | | | |
| 3 | SCA | 6 | | | | | |
| 3 | SCA | 7 | | | | | |
| 3 | SCA | 8 | | | | | |
| 3 | SCA | 9 | | | | | |
| 3 | SCA | 10 | | | | | |
| 3 | SCA | 11 | | | | | |
| 3 | SCA | 12 | | | | | |
| 3 | CCA | 0 | 13.41 | 0.1186 | 13.24 | 2.3920 | 105 |
| 3 | CCA | 1 | 15.68 | 0.0272 | 15.28 | 1.3632 | 2561 |
| 3 | CCA | 2 | 17.30 | 0.0254 | 17.32 | 1.4548 | 3336 |
| 3 | CCA | 3 | 18.11 | 0.0472 | 17.91 | 1.2768 | 787 |
| 3 | CCA | 4 | 18.72 | 0.1315 | 18.85 | 1.8896 | 87 |
| 3 | CCA | 5 | | | | | |
| 3 | CCA | 6 | | | | | |
| 3 | CCA | 7 | | | | | |
| 3 | CCA | 8 | | | | | |
| 3 | CCA | 9 | | | | | |
| 3 | CCA | 10 | | | | | |
| 3 | CCA | 11 | | | | | |

| | | Model Estimation | | | Observed Values | | |
|---|---|---|---|---|---|---|---|
| **Quarter** | **Area** | **Age (years)** | **Mean Length (cm)** | **SE** | **Mean Length (cm)** | **sd** | **N** |
| 3 | CCA | 12 | | | | | |
| 3 | ORWA | 0 | | | | | |
| 3 | ORWA | 1 | 16.08 | 0.0615 | 16.89 | 2.2354 | 452 |
| 3 | ORWA | 2 | 18.29 | 0.0317 | 18.58 | 1.8388 | 1813 |
| 3 | ORWA | 3 | 19.68 | 0.0226 | 19.73 | 1.3934 | 4304 |
| 3 | ORWA | 4 | 20.29 | 0.0210 | 20.14 | 1.4359 | 5412 |
| 3 | ORWA | 5 | 20.82 | 0.0234 | 20.65 | 1.5636 | 3832 |
| 3 | ORWA | 6 | 21.29 | 0.0305 | 21.27 | 1.6711 | 1946 |
| 3 | ORWA | 7 | 21.72 | 0.0463 | 21.94 | 1.6329 | 746 |
| 3 | ORWA | 8 | 22.29 | 0.0692 | 22.74 | 1.4219 | 319 |
| 3 | ORWA | 9 | 22.63 | 0.0949 | 23.05 | 1.3300 | 167 |
| 3 | ORWA | 10 | 22.90 | 0.1519 | 23.44 | 1.2956 | 64 |
| 3 | ORWA | 11 | 23.02 | 0.1872 | 23.36 | 1.1438 | 42 |
| 3 | ORWA | 12 | 23.41 | 0.2474 | 23.54 | 0.9771 | 24 |
| 4 | CANADA | 0 | | | | | |
| 4 | CANADA | 1 | | | | | |
| 4 | CANADA | 2 | | | | | |
| 4 | CANADA | 3 | 20.05 | 0.2626 | 19.76 | 1.7626 | 25 |
| 4 | CANADA | 4 | 22.55 | 0.1746 | 22.17 | 1.0619 | 58 |
| 4 | CANADA | 5 | 23.23 | 0.1538 | 22.80 | 0.6737 | 76 |
| 4 | CANADA | 6 | 23.96 | 0.1553 | 23.36 | 0.7688 | 74 |
| 4 | CANADA | 7 | 24.62 | 0.1698 | 23.66 | 0.6258 | 62 |
| 4 | CANADA | 8 | 25.00 | 0.2929 | 23.85 | 0.7452 | 20 |
| 4 | SCA | 0 | 13.57 | 0.0597 | 12.21 | 1.4530 | 645 |
| 4 | SCA | 1 | 16.37 | 0.0363 | 15.37 | 1.9041 | 2009 |
| 4 | SCA | 2 | 17.65 | 0.0407 | 17.34 | 2.1331 | 1302 |
| 4 | SCA | 3 | 18.26 | 0.0708 | 18.06 | 1.9861 | 422 |
| 4 | SCA | 4 | 19.52 | 0.1731 | 19.58 | 2.2035 | 57 |
| 4 | SCA | 5 | | | | | |
| 4 | SCA | 6 | | | | | |
| 4 | SCA | 7 | | | | | |
| 4 | SCA | 8 | | | | | |
| 4 | CCA | 0 | 14.45 | 0.0965 | 13.96 | 2.4492 | 220 |
| 4 | CCA | 1 | 16.69 | 0.0413 | 15.35 | 1.3972 | 2253 |
| 4 | CCA | 2 | 18.74 | 0.0412 | 17.33 | 1.4494 | 2371 |
| 4 | CCA | 3 | 20.20 | 0.0537 | 19.35 | 1.6792 | 972 |

| | | Model Estimation | | | Observed Values | | |
|---|---|---|---|---|---|---|---|
| **Quarter** | **Area** | **Age (years)** | **Mean Length (cm)** | **SE** | **Mean Length (cm)** | **sd** | **N** |
| 4 | CCA | 4 | 21.69 | 0.0947 | 21.11 | 1.7569 | 215 |
| 4 | CCA | 5 | 23.00 | 0.1608 | 22.51 | 1.5692 | 68 |
| 4 | CCA | 6 | | | | | |
| 4 | CCA | 7 | | | | | |
| 4 | CCA | 8 | | | | | |
| 4 | ORWA | 0 | | | | | |
| 4 | ORWA | 1 | 17.46 | 0.1123 | 16.36 | 2.1377 | 166 |
| 4 | ORWA | 2 | 19.80 | 0.1214 | 18.71 | 2.1707 | 124 |
| 4 | ORWA | 3 | 20.68 | 0.1060 | 19.58 | 2.0005 | 165 |
| 4 | ORWA | 4 | 21.66 | 0.1190 | 21.02 | 1.7426 | 135 |
| 4 | ORWA | 5 | 22.25 | 0.1353 | 21.63 | 1.2225 | 101 |
| 4 | ORWA | 6 | 22.89 | 0.1505 | 22.15 | 1.0803 | 80 |
| 4 | ORWA | 7 | 23.74 | 0.2433 | 23.03 | 0.9814 | 29 |
| 4 | ORWA | 8 | | | | | |

Empirical mean length-at-age and standard deviation along with model estimated mean age and standard error are given. Length bins with 20 or fewer fish were not included in the analysis.
